# Ferroptosis open a new door for colorectal cancer treatment

**DOI:** 10.3389/fonc.2023.1059520

**Published:** 2023-03-16

**Authors:** Hong Liang, Xia He, Yitong Tong, Niuniu Bai, Yushu Pu, Ke Han, Yi Wang

**Affiliations:** ^1^ Department of Pharmacy, Sichuan Academy of Medical Sciences & Sichuan Provincial People’s Hospital, School of Medicine, University of Electronic Science and Technology of China, Chengdu, China; ^2^ Personalized Drug Therapy Key Laboratory of Sichuan Province, School of Medicine, University of Electronic Science and Technology of China, Chengdu, China; ^3^ Chengdu Second People’s Hospital Party Committee Office, Chengdu, China; ^4^ School of Pharmacy, Shanxi Medical University, Taiyuan, China; ^5^ Nanchang University Queen Mary School, Nanchang, China; ^6^ Department of Pharmacy, The First People’s Hospital of Chengdu, Chengdu, China; ^7^ Department of Critical Care Medicine, Sichuan Academy of Medical Science and Sichuan Provincial People's Hospital, University of Electronic Science and Technology of China, Chengdu, Sicuhan, China

**Keywords:** ferroptosis, colorectal cancer, molecular mechanism, inducer, nanomedicine 1

## Abstract

Colorectal cancer (CRC) is the third highest incidence and the second highest mortality malignant tumor in the world. The etiology and pathogenesis of CRC are complex. Due to the long course of the disease and no obvious early symptoms, most patients are diagnosed as middle and late stages. CRC is prone to metastasis, most commonly liver metastasis, which is one of the leading causes of death in CRC patients. Ferroptosis is a newly discovered cell death form with iron dependence, which is driven by excessive lipid peroxides on the cell membrane. It is different from other form of programmed cell death in morphology and mechanism, such as apoptosis, pyroptosis and necroptosis. Numerous studies have shown that ferroptosis may play an important role in the development of CRC. For advanced or metastatic CRC, ferroptosis promises to open a new door in the setting of poor response to chemotherapy and targeted therapy. This mini review focuses on the pathogenesis of CRC, the mechanism of ferroptosis and the research status of ferroptosis in CRC treatment. The potential association between ferroptosis and CRC and some challenges are discussed.

## 1 Introduction

CRC is a common gastrointestinal malignancy, CA: A Cancer Journal for Clinicians usually combines colon, rectum, and anus cancers as CRC for statisticsor. The latest data showed that in 2020, there were more than 1.9 million new cases of CRC and about 935,000 deaths in the world. The incidence and mortality of CRC ranked the third (10.0%) and second (9.4%) worldwide, respectively (1). The National Cancer Center also released the cancer report in 2022. The data showed that there were about 560,000 new cases and 290,000 deaths of CRC in China. Incidence and mortality ranked second (12.2%) and fifth (9.5%) in China, respectively (2). CRC has posed a major threat to human health. The 5-year survival rate for advanced CRC is only 14% (3). Due to the unsatisfactory effects of traditional chemotherapeutic drugs or targeted drugs, it is urgent to explore new treatment strategies. Ferroptosis is a research hotspot in recent years. It has a unique mechanism that distinguishes it from other cell death manners. More importantly, studies have found that ferroptosis is closely related to multiple cancer including CRC, which can open a new door for CRC treatment.

## 2 Colorectal cancer

### 2.1 Symptoms and etiology

**Figure 1:**
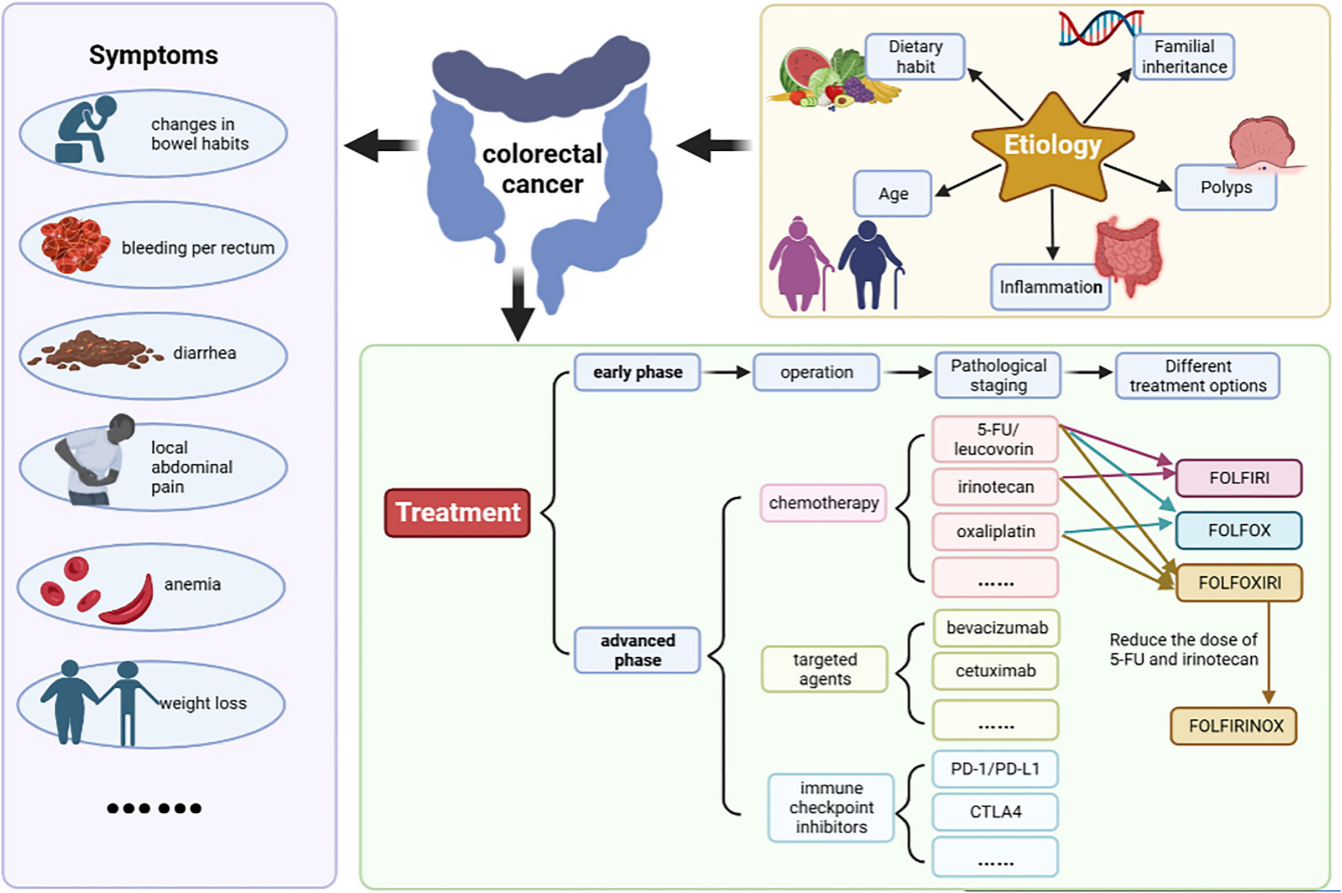
The symptoms, etiology and treatment of CRC. The symptoms include changes in bowel habits, bleeding per rectum, diarrhea, local abdominal pain, anemia and weight loss etc. At present, the etiology of CRC is not completely clear. It is generally believed that dietary habit, familial inheritance, polyps, inflammation, age are closely related to the disease. Surgery is the preferred treatment for patients with early-stage CRC. Commonly used chemotherapy drugs mainly include 5-fluorouracil (5-FU)/leucovorin (LV), irinotecan, and oxaliplatin etc. Targeted therapeutic drugs mainly include cetuximab and bevacizumab. Basic chemotherapy regimens included FOLFIRI (irinotecan+5-FU+LV), FOLFOX (oxaliplatin+5-FU+LV), FOLFOXIRI (oxaliplatin+irinotecan+5-FU+LV) and its modified regimen FOLFIRINOX (reduced doses of irinotecan and 5-FU).

The symptoms of CRC are not obvious in the early stage. As the tumor grows, it shows symptoms such as changes in bowel habits, bleeding per rectum, diarrhea, local abdominal pain, anemia and weight loss (**Figure 1**) (4). CRC can occur anywhere in the colon or rectum, but the sigmoid colon and rectum are the most common case (5). At present, the etiology of CRC is not completely clear. It is generally believed that the following factors are closely related to the disease (**Figure 1**).

#### 2.1.1 Dietary habit

It mainly includes the intake of high fat, high protein, low dietary fiber and nitrite compounds; vitamin deficiency; intestinal flora imbalance and other factors. Obesity induced by high fat diet has been a high risk factor for CRC.

High fat diet not only can stimulate the increase of bile secretion, but also promotes the growth of some anaerobic bacteria in the intestine. Bile alcohol and bile salt are decomposed by anaerobic bacteria to form unsaturated cholesterol, such as deoxycholic acid and lithocholic acid, which is carcinogens or co-carcinogens (6). Studies have shown that high fat diet can reduce the expression of the major histocompatibility complex class II (MHC class II) genes in intestinal epithelial cells (including intestinal stem cells), thereby disrupting the intestinal flora and promoting intestinal tumorigenesis (7).

High protein diet can increase harmful metabolites in the gut, such as N-nitroso compounds (NOCs) and H_2_S. NOCs can lead to DNA alkylation, which can induce genetic mutations that ultimately lead to cancer (8, 9). After ingestion, protein is decomposed into various absorbable amino acids including methionine and cysteine that can be further decomposed by sulfate-reducing bacteria to produce H_2_S, which can promote the occurrence of CRC by inhibiting butyrate oxidation, destroying the intestinal barrier, and interacting with ROS to induce DNA damage (10, 11).

Dietary fiber can increase intestinal peristalsis, promote stool excretion, and reduce the food transit time in the colon, thus reducing the contact time of potential carcinogens with intestinal mucosa (12, 13). Moreover, the residues of dietary fiber can be fermented by intestinal microbiota to produce short chain fatty acids (SCFA), especially butyrate, which has important physiological effects. Butyrate is not only the main energy source of intestinal microbiota and intestinal epithelial cells, but also plays an important role in colon health by inhibiting cell proliferation, inducing cell differentiation, promoting cell apoptosis, and reducing tumor cell invasiveness (14, 15). Therefore, lack of dietary fiber will increase the risk of intestinal diseases.

### 2.1.2 Familial inheritance

Approximately 20-30% of CRC cases are associated with genetic factors, 2-5% of CRC is caused by inherited syndrome, mainly including Lynch syndrome, familial adenomatous polyposis, juvenile polyposis syndrome, *MUTYH*-associated polyposis, and Peutz-Jeghers syndrome etc (16, 17).

### 2.1.3 Polyps

The incidence of CRC is closely related to polyps. Colorectal polyps including adenomatous polyps (adenomas), hamartomatous polyps and serrated polyps are early lesions of cancer (18). It takes at least 10 years for the progression of “polyp-adenomas-CRC” (19). The formation factors of polyps mainly include genetic factors, obesity, smoking, drinking, age and so on (20). Diet affects the development of polyps and inflammation. Studies have reported that high fat and protein (especially red meat) intake can significantly increase intestinal polyp formation and inflammation (21–23). However, the intake of dietary fiber can promote stool excretion, reduce the contact of carcinogens with the intestinal mucosa, and produce short-chain fatty acids, thereby reducing the formation of intestinal polyps and inflammatory response (24).

#### 2.1.4 Inflammation

Chronic inflammatory stimulation can lead to the development of CRC. Inflammatory bowel disease (IBD) mainly includes ulcerative colitis and Crohn’s disease, which is one of the high risk factors for the development of CRC (25). Compared with the general population, patients with IBD have an approximately 2-6 folds increased risk of developing CRC (26).

#### 2.1.5 Age

Population aging has a significant impact on the incidence of CRC. Surveys show that the incidence of CRC worldwide increases with age. In the United States, the incidence of CRC is three times higher in people over 65 years old than in 50-64 years old, and approximately 30 times higher than in 25-49 years old (27).

### 2.2 Pathogenesis

The pathogenesis of CRC is a multi-gene, multi-step and multi-pathway process, which is caused by the gradual accumulation of complex genetic and epigenetic events. CRC can be divided into three classifications by etiology: sporadic CRC (~80% cases), hereditary CRC (~20% cases), and inflammatory CRC (<2% cases) (28). The most classic pathological process is the progression from polyps to adenomas and finally to adenocarcinoma. Intestinal polyps are benign precursors of most CRC. Adenomas and serrated polyps are the two main subtype. Approximately 85-90% of sporadic CRCs develop from adenomas. The serrated polyps pathway accounts for only 10-15% of sporadic CRC (29).

**Figure 2:**
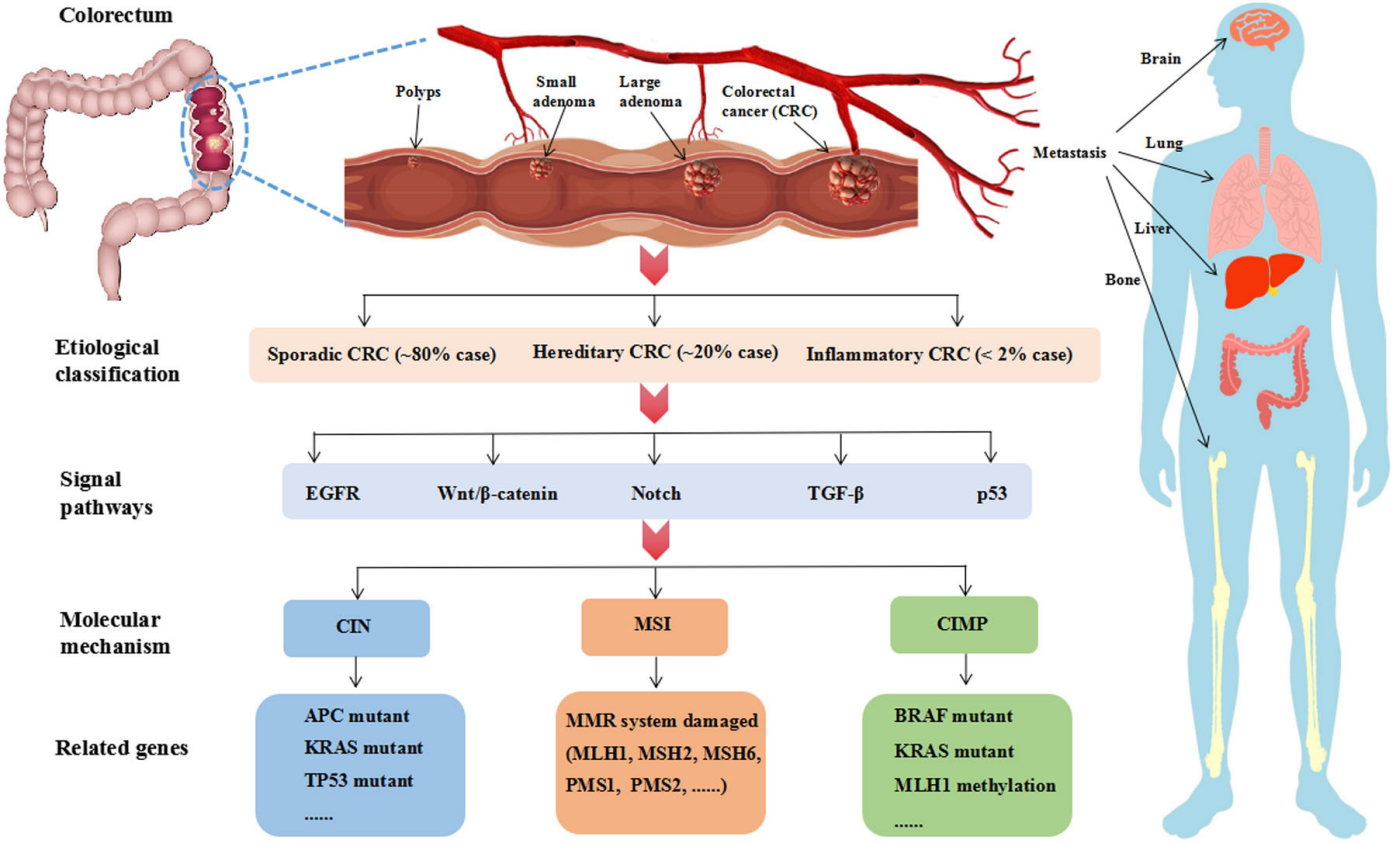
The pathological process, classification, signal pathway and molecular mechanism of CRC. The classic pathological process of CRC is “polyps-adenoma-adenocarcinoma”, which needs over 10 years. Advanced CRC is prone to metastasis. Liver metastasis is the most common CRC more than 50%. According to the etiology, CRC can be divided into three classifications. Sporadic CRC is the most common clinical case accounting for approximately 80%. Hereditary CRC is associated with genetic factors accounting for approximately 20%, in which 2-5% cases are caused by inherited syndrome such as Lynch syndrome and familial adenomatous polyposis. Inflammatory CRC is rare in clinical, which is less than 2%. As reports, EGFR, Wnt/β-catenin, Notch, TGF-β and p53 are the main signal pathways at present. And CIN, MSI, CIMP are the major molecular mechanism. CIN is related to high gene mutant of APC, KRAS and TP53. MSI is driven by the impairment of DNA mismatch repair (MMR) system. CIMP is a form of epigenetic modification and relates to the hypermethylation of CpG islands. CIMP-high subtype is BRAF mutations and MLH1 methylation and CIMP-low subtype is KRAS mutations.

It is currently believed that the pathogenesis of CRC is mainly related to the abnormality of five cell signal pathways: epidermal growth factor receptor (EGFR) signal pathway (including MAPK and PI3K signal pathway), Wnt/β-catenin signal pathway, Notch signal pathway, p53 signal pathway and Transforming growth factor-beta (TGF-β) signal pathway (30). Further analysis shows that the molecular mechanism of CRC can be divided into three type (**Figure 2**): chromosomal instability (CIN), microsatellite instability (MSI) and CpG island methylation phenotype (CIMP) (31). These three pathways are not mutually exclusive.

#### 2.2.1 CIN mechanism

The CIN is the most classical pathway, accounting for 80-85% of all CRC cases. Aneuploidy or structural chromosomal abnormalities, frequent loss of heterozygosity at tumor suppressor loci, and chromosomal rearrangement are the main features (32). These changes will affect important genes related to the maintenance of cell function, such as adenomatous polyposis coli (APC), KRAS and TP53. About 80% of CRC patients have APC mutations, which can active Wnt signal pathway. There are three characteristic genes related to human tumors in RAS gene family including HRAS, NRAS and KRAS. Among them, KRAS is the most mutated in 40% of sporadic CRC (33). KRAS mutations can active MAPK and PI3K signaling pathway, which increases cell proliferation (34). It is well known that p53 is a tumor suppressor protein, which is encoded by the TP53 gene. TP53 mutations occurs in 40-50% of sporadic CRC, which is a key step driving the development of CRC (35).

#### 2.2.2 MSI mechanism

Microsatellite is short nucleotide tandem repeat in the DNA sequence, which is easy to get a replication error due to repeated structure. Mismatch repair (MMR) system mainly including MLH1, MSH2, MSH6, PMS1 and PMS2 proteins can repair these errors. MSI is driven by the loss of function of MMR system, and promoter hypermethylation is considered to be the main cause of gene silencing. MSI accounts for about 15% of sporadic CRC. Moreover, germ-line mutations in these genes causes Lynch syndrome, which is the most common inherited type of CRC (36).

#### 2.2.3 CIMP mechanism

Site specific DNA hypermethylation of CG dinucleotides (CpG islands) associated with promoters of tumor suppressor genes and DNA repair genes leads to transcriptional silencing that promotes cancer initiation and progression. CIMP is first identified in CRC, which is related to the hypermethylation of CpG islands in the promoter region of tumor suppressor genes such as MINT1, MINT2, MINT3 and MLH1 (37, 38). It is a form of epigenetic modification and can be divided into two type. CIMP-high subtype is BRAF mutations and MLH1 methylation. CIMP-low subtype is KRAS mutations. Studies have shown that BRAF and KRAS direct the assembly of distinct corepressor complexes on a common promoter through different pathways leading to promoter hypermethylation and transcriptional silencing of CIMP (39). BRAF increases phosphorylation of MAFG (a small MAF protein) *via* the MEK/ERK signaling pathway, thereby protecting MAFG from polyubiquitination and subsequent proteasome-mediated degradation. Subsequently, MAFG recruits corepressor complexes including BACH1, CHD8 and DNMT3B, resulting in promoter hypermethylation and transcriptional silencing of CIMP (40). KRAS increases the level of zinc finger protein 304 (ZNF304) by transcriptionally upregulating the serine/threonine kinase protein kinase D1 (PRKD1) and ubiquitin-specific peptidase 28 (USP28). Subsequently, ZNF304 recruits corepressor complexes including KAP1, SETDB1 and DNMT1, resulting in promoter hypermethylation and transcriptional silencing of CIMP (41). CIMP is highly associated with the CRC developing from serrated polyps pathway, but the underlying mechanism remains to be further explored.

### 2.3 Metastasis and treatment of CRC

CRC treatment mainly involves surgery, chemotherapy and radiotherapy. According to clinical guidelines, surgery is the preferred treatment for patients with early-stage CRC, and chemotherapy or targeted therapy should be used for patients with advanced or metastatic CRC (42). Commonly used chemotherapy drugs mainly include 5-fluorouracil (5-FU)/leucovorin (LV), irinotecan, oxaliplatin, capecitabine, trifluridine, tippiridine and raltitrexed. Targeted therapeutic drugs mainly include cetuximab, bevacizumab, regorafenib, panitumumab and so on. Clinically, it is mainly a comprehensive treatment based on chemotherapy. Basic chemotherapy regimens includes FOLFIRI (irinotecan+5-FU+LV), FOLFOX (oxaliplatin+5-FU+LV), FOLFOXIRI (oxaliplatin+irinotecan+5-FU+LV) and its modified regimen FOLFIRINOX (reduced doses of irinotecan and 5-FU) (**Figure 1**) (43). The latest NCCN guidelines (version 1. 2022) recommended FOLFIRINOX instead of FOLFOXIRI because the FOLFOXIRI regimen at high doses of 5-FU showed greater toxicity in American patients.

Surgery is the most effective way to cure CRC (44). However, the surgical cure rate and 5-year overall survival rate of CRC have been hovering around 50% (45), and the main reasons for treatment failure are the high recurrence and metastasis rates.

CRC is prone to metastasis. It usually occurs in the liver, lung and abdominal cavity. In addition, bone and brain metastasis may occur. Among them, liver metastasis is the most common CRC, and the rate of liver metastasis can over 50%. It is worth noting that 15-25% of CRC patients appeared liver metastasis at initial diagnosis (46). Without any treatment, most people with liver metastasis survive only a few months. Liver metastasis is considered as the leading direct cause of CRC-related death. It is fewer than 20% of 5-year survival rate for metastatic CRC patients (47). Due to the clinical benefit rate remaining at a low level, it is urgent to explore new treatment strategies for CRC. Ferroptosis is a novel manner of programmed cell death, which plays an important role in multiple diseases, such as neurological diseases, liver diseases, gastrointestinal diseases and cancer (48, 49).

## 3 Ferroptosis

### 3.1 Characteristics of ferroptosis

Programmed cell death is an active and orderly way of cell death determined by genes, which is closely related to the maintenance of life homeostasis and the occurrence of diseases, mainly including apoptosis, pyroptosis, necrosis and autophagy (50, 51). Cellular ferroptosis is a term coined in 2012 by Dixon et al. It is referred to induce cell membrane breakage through excess lipid peroxides on cell membranes, a process in which iron is involved in regulation (52). Ferroptosis is different from other type of cell death in morphology and mechanism, and its main characteristics are as follows.

#### 3.1.1 Morphological characteristics

The main features are decreased mitochondrial volume, decreased or disappearance crista, increased mitochondrial membrane density, disruption of mitochondrial membrane and without chromatin condensation. However, the nucleus structure is intact and morphological changes are not obvious (53).

#### 3.1.2 Biological characteristics

It is mainly manifested in the increase of reactive oxygen species (ROS), the aggregation of iron ions, the increase of oxidation level of reduced coenzyme II (NADPH), the inhibition of glutathione peroxidase 4 (GPX4) and cystine/glutamic acid antiporter transporter (system Xc^-^) (54).

#### 3.1.3 Immunological characteristics

Ferroptosis not only affects innate immunity by affecting the number and function of immune cells (e.g. macrophages and neutrophils) but also affects adaptive immunity through triggering inflammatory or specific responses after ferroptotic cells recognized by immune cells (e.g. T and B lymphocytes). The death of immune cells caused by ferroptosis may impair the immune response. In contrast, non-immune cell death caused by ferroptosis can activate damage-associated molecular patterns (DAMP), thereby activating immune responses (55).

#### 3.1.4 Genetic characteristics

The genes/proteins that regulate ferroptosis can be mainly divided into three categories, among which SLC7A11, SLC3A2, ATF3, p53, etc. mainly regulate cystine uptake; GPX4, ACSL4, LPCAT3, FSP1, NRF2, etc. mainly regulate lipid metabolism; TFR1, HSPB1, IREB2, NFS1 and others mainly regulate iron metabolism (56).

### 3.2 Mechanism of ferroptosis

**Figure 3:**
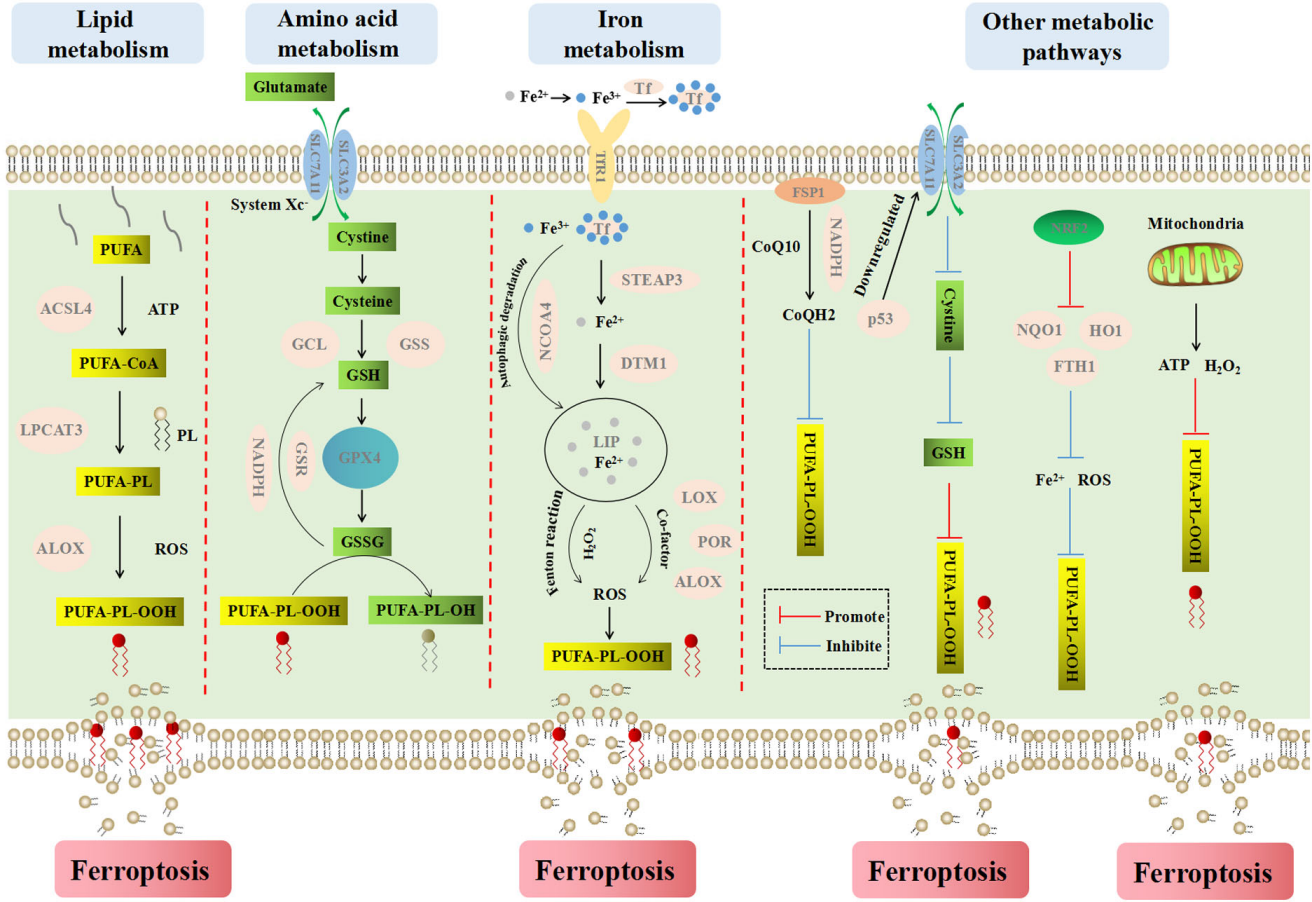
The main metabolic pathways and processes of ferroptosis. Ferroptosis is a iron-dependent cell death type, which causes cell membrane rupture by lipid peroxidesn. Lipid metabolism explains the formation process of lipid peroxides from PUFA to PUFA-PL-OOH. Amino acid metabolism explains the process of cell inhibiting lipid peroxidation by GSH. Iron metabolism explains the important role of iron ions for inducing ferroptosis. Fe^2+^ reacts with endogenous H_2_O_2_ in Fenton reaction to produce abundant ROS, which can promote lipid peroxidation and ferroptosis. Other metabolism ways mainly explain the influence of FSP1, NADPH, CoQ10, p53, NRF2 and mitochondria on ferroptosis.

The occurrence and execution of ferroptosis involve multiple metabolic pathways and processes, including lipid metabolism, amino acid metabolism, iron metabolism, and other metabolic pathways (**Figure 3**). Each aspect does not exist independently, but influences and penetrates each other.

#### 3.2.1 Lipid metabolism

Lipid peroxidation is key to the occurrence of ferroptosis. Compared with monounsaturated fatty acids, polyunsaturated fatty acids (PUFA) have diallyl moiety. Therefore, phospholipids containing polyunsaturated fatty acids (PUFA-PL) are very susceptible to peroxidation (57). The peroxidation of PUFA-PL is mainly catalyzed by non-enzymatic autooxidation, which is actuated by Fenton reaction and catalyzed by iron. Lipid peroxides can be formed by capturing an unstable hydrogen atom in diallyl group on PUFA-PL (58).

Acyl-coa synthetase long-chain family member 4 (ACSL4) can catalyze the conversion of long-chain fatty acids to fatty acyl-coenzymes in an ATP-dependent manner, which plays a crucial role in the process of iron-dependent oxidative stress (59). ACSL4 tends to bind long-chain polyunsaturated fatty acids such as arachidonic acid (AA) and adrenic acid (ADA), and then specifically esterifies PUFA to PUFA-CoA such as AA-CoA and ADA-CoA under the action of acetyl coenzyme A (CoA). Then, under the catalysis of lysophosphatidylcholine acyltransferase 3 (LPCAT3), PUFA-CoA combines with phospholipids on the cell membrane to form PUFA-PL, which is peroxidized to PUFA-PL-OOH under the regulation of dioxygenase (ALOX) (60–62). On the one hand, the accumulation of phospholipid peroxides makes the cytomembrane thin and increases its curvature, and the accelerated influx of oxidants intensifies the oxidation, which eventually leads to membrane perforation, rupture and the release of cellular contents (63). On the other hand, lipid peroxides are further broken down into active substances that can deplete nucleic acids and proteins, or accelerate the destruction of membrane structural integrity, leading to cell ferroptosis (64, 65).

#### 3.2.2 Amino acid metabolism

System Xc^-^ is a sodium independent transmembrane cystine/glutamate antiporter located on the plasma membrane. It belongs to heteromeric amino acid transporters (HATs), which are composed of the light chain subunit SLC7A11 (xCT) and the heavy chain subunit SLC3A2 (4F2hc) linked by covalent disulfide bonds. SLC7A11 is a member of the SLC7 family, which mainly includes two subfamilies of cationic amino acid transporters (CATs, SLC7A1-4 and SLC7A14) and L-type amino acid transporters (LATs, SLC7A5-13 and SLC7A15) (66). LATs can bind specifically to two members of SLC3 (SLC3A1 and SLC3A2) to form HATs (67).

The activity of system Xc^-^ is mainly determined by SLC7A11, which is highly specific to cystine and glutamate. It can pump glutamate out of the cell in a 1:1 ratio while transferring extracellular cystine into the cell (68). Excessive release of glutamate will increase the concentration of extracellular glutamate, which in turn regulates the function of the system Xc^-^, reducing the intake of cystine and the excretion of glutamate. At the same time, released glutamate is an excitatory neurotransmitter with dual effects of neurotoxicity and excitabilit (69).

The cystine that enters the cell is decomposed into cysteine in a highly reduced environment. Then cysteine is converted to GSH under the catalysis of glutamate cysteine ligase (GCL) and glutathione synthetase (GSS) (70). GPX4 is a kind of intracellular antioxidant enzymes, which can catalyze the conversion of GSH into its oxidized form GSSG, and convert harmful lipid peroxide (L-OOH) into non-toxic lipid alcohol (L-OH), so as to inhibit lipid peroxidation and prevent the occurrence of ferroptosis. With the help of glutathione reductase (GSR), excess GSSG is reduced to GSH by NADPH and enters the next cycle. On the contrary, when system Xc^-^ or GPX4 is inhibited or inactivated, the redox balance in the cell is dysregulated, which will promote the ferroptosis (71).

#### 3.2.3 Iron metabolism

Fe^2+^ and Fe^3+^ are two oxidation states of iron. Iron bines with transferrin in blood circulation and exists in the form of Fe^3+^. After entering cells *via* the transferrin receptor 1 (TfR1) on the cell membrane, Fe^3+^ is deoxidized and converted to Fe^2+^ under the action of iron oxide reductase six transmembrane epithelial antigen of the prostate 3 (STEAP3) (72). After that, Fe^2+^ is transported to the labile iron pool (LIP) in the cytoplasm by divalent metal transporter 1 (DMT1) (73). Intracellular iron is mainly stored in ferritin, and the autophagic degradation of ferritin, which is mediated by nuclear receptor coactivator 4 (NCOA4) (74), can release iron into LIP. Thus, blocking NCOA4 can reduce the level of LIP and inhibit ferroptosis (75). In contrast, enhanced ferritin phagocytosis can increase the LIP and promote ferroptosis. Dynamic iron pools can maintain iron balance under normal physiological conditions while Fe^2+^ accumulates in cells under pathological conditions. Excessive Fe^2+^ can not only react with endogenous H_2_O_2_ in Fenton reaction to produce a large amount of ROS, but also enhance the activities of multiple metabolic enzymes (such as LOXs, PDH1, ALOX and POR) with iron as a co-factor to promote the generation of ROS (76). ROS can promote the peroxidation of PUFA-PL on the cell membrane to generate lipid peroxides and cause ferroptosis. Therefore, factors related to iron metabolism are potential target sites for inducing ferroptosis.

#### 3.2.4 Other metabolic pathways

Several metabolic processes in mitochondria are also significant in triggering ferroptosis. Mitochondria are the main sites for the production of intracellular ROS, which is critical for lipid peroxidation and ferroptosis. Complexes I, II and III in mitochondria are mainly located in the respiratory chain and generate superoxide, which is then converted to H_2_O_2_ by superoxide dismutase (77). H_2_O_2_ reacts with labile iron to generate hydroxyl radicals (**·**OH) and drives PUFA-PL peroxidation through Fenton reaction. In addition, electron transport and proton pumps in mitochondria are vital to produce ATP, which also contributes to ferroptosis (78). When ATP is depleted, PUFA-PL and ferroptosis are inhibited by activation of AMP-activated protein kinase (AMPK) and inactivation of acetyl-CoA-carboxylaze (ACC). In contrast, in the condition of sufficient energy, PUFA-PL synthesis and ferroptosis are promoted (79).

Other factors affecting ferroptosis include coenzyme Q10 (CoQ10), NADPH, p53, nuclear factor E2 related factor 2 (NRF2), etc. Ferroptosis suppressor protein 1 (FSP1) is localized to the plasma membrane and acts as an NADPH dependent oxidoreductase that reduces ubiquinone CoQ10 to ubiquinol (CoQH2) to prevent lipid oxidation and inhibits ferroptosis (80, 81). p53 can inhibit cystine uptake and ferroptosis by down-regulating the expression of SLC7A11 (82). NRF2 plays a significant role in maintaining intracellular redox equilibrium. It up-regulates the level of many genes (NQO1, HO1 and FTH1, etc.) involving in the metabolism of iron and ROS through the p62-Keap1-NRF2 pathway, then inhibits ferroptosis (83).

## 4 Research status of ferroptosis in CRC

### 4.1 Potential association between ferroptosis and CRC

#### 4.1.1 The role of ferroptosis in liver disease

Liver metastasis is the most common cause of CRC, and the liver is a favorable site for ferroptosis. Liver disease is currently the most studied type of ferroptosis. On the one hand, programmed cell death triggered by lipid accumulation in hepatocytes is considered as a possible cause of liver tissue damage and inflammation (84). On the other hand, the liver is the main organ of iron deposition, and the dysregulation of iron metabolism leads to the production of a large amount of free iron, which can significantly increase the sensitivity of hepatic cells to ferroptosis (85). We know that nonalcoholic steatohepatitis (NASH) is a severe chronic liver disease characterized by lipid droplet accumulation, hepatocyte death, and inflammatory cell infiltration. It is an important risk factor for liver cirrhosis and carcinogenesis. Studies have shown that ferroptosis is the preferential form of cell death in NASH. Liver tissue inflammation is significantly inhibited by specific inhibition of ferroptosis (86), thereby reducing the possibility of CRC.

#### 4.1.2 The gastrointestinal tract provides favorable conditions for ferroptosis

In fact, dietary fat is decomposed into fatty acids and monoacylglycerols by pancreatic lipase in the intestinal cavity. Fatty acids penetrate the mucus layer on the surface of microvilli and are absorbed by villous intestinal epithelial cells (CD36, FABP, etc.) then transport to the endoplasmic reticulum (87). With the action of multiple enzymes such as fatty acid synthase, long-chain fatty acids and triglycerides are resynthesized, which participates in the formation of cell membranes or reserve energy for the body (88). Based on studies of ferroptosis in liver disease, we can make reasonable assumptions. Since the gastrointestinal tract is the primary site for the metabolism and absorption of nutrients, including fat and iron, it provides favorable conditions for ferroptosis of gastrointestinal cells (89).

#### 4.1.3 CRC tumor suppressor p53 regulates ferroptosis

In addition, in sporadic CRC, p53 is mutated in the late stage of carcinogenesis, which promotes the further progression of adenoma to adenocarcinoma (90). In colitis associated CRC, p53 is more important and the mutating early can initiate tumorigenesis (91). As the most important tumor suppressor gene, p53 can not only cause apoptosis, cell cycle arrest and senescence, but also play a tumor suppressor function by inducing ferroptosis as described above.

#### 4.1.4 Ferroptosis and CRC prognosis

There may be a correlation between ferroptosis and prognosis of CRC. Biomarkers of ferroptosis, including GPX4, NOX1 and ACSL4, are important prognostic markers in CRC. Moreover, IFN- γ may be involved in tumor death, suggesting that patients with CRC have a good prognosis (92). Obesity is closely related to poor prognosis in patients with advanced CRC, because fat-derived exosomes reduce the susceptibility to ferroptosis in CRC and promote chemotherapy resistance to oxaliplatin (93).

### 4.2 Medications associated with CRC ferroptosis

#### 4.2.1 Ferroptosis inducers

Are ROS-promoting compounds effective in inducing ferroptosis? We knew that endogenous ROS were mainly formed in the mitochondrial respiratory chain, and low levels of ROS played an important role in regulating the biological functions of cells. However, excessive ROS could oxidize proteins, lipids and DNA, break redox homeostasis, and lead to cell death. ROS-induced lipid peroxidation could induce programmed cell death in the form of apoptosis, autophagy and ferroptosis.

Firstly, the formation of lipid peroxides can be achieved through different pathways, mainly including non-enzymatic and enzymatic pathways. The non-enzymatic pathway was an iron-dependent process and an important link in the formation of ferroptosis. As the name implied, the formation of lipid peroxides by lipoxygenase catalysis was the enzymatic pathway. Secondly, lipid peroxides could initiate different forms of programmed cell death through different signaling pathways. They could not only initiate apoptosis through pathways such as NF-κB, MAPK and PKC, but also initiate autophagy through AMPK/mTORC and JNK-Bcl-2/Beclin 1 (94). For example, knockdown of protocadherin 7 (PCDH7) affects autophagy and induces ferroptosis, which enhances the sensitivity of colon cancer cells to chemotherapy by inhibiting the MEK1/2/ERK/c-FOS axis (95). In addition, they could achieve ferroptosis by interacting directly with the cell membrane and disrupting membrane integrity. Therefore, ferroptosis was different from other forms of cell death in cell morphology and mechanism.

The changes of ferrous ion concentration, lipid peroxides and ferroptosis-related proteins were the key indicators to identify the occurrence of ferroptosis (64). For example, Liu et al. demonstrated that oxaliplatin could promote ferroptosis by inhibiting the NRF2 signaling pathway. The results showed that after HT29 cells were treated with oxaliplatin, the Fe^2+^ concentration in the cells was significantly increased and the ferroptosis-related protein GPX was significantly decreased, thus contributing to the production of a large amount of ROS and lipid peroxides (95). Dichloroacetic acid (DCA) can cause iron death in colorectal cancer stem cell (CSC), manifested as increased iron concentrations, lipid peroxides, and glutathione levels (96).

Although many chemotherapeutic drugs could induce ROS production, there was no significant change in the ferroptosis-related characteristics during cell death. Therefore, it did not belong to ferroptosis. They may cause the destruction of proteins or DNA by ROS or the production of lipid peroxides by enzymatic pathways, thereby causing cell death through apoptosis and autophagy. For example, Liu et al. found that the small molecule compound VB1 (extracted from *Vitex negundo*) showed anti-tumor activity, which led to DNA damage and apoptosis by promoting the accumulation of ROS in cells (97).

Ferroptosis inducers (FIN) are compounds that can induce ferroptosis in cells. In tumor therapy, they can be mainly divided into two kinds according to their mechanism of action. 1) *FIN associated with System Xc^-^.* These inducers act primarily by inhibiting SLC7A11-mediated cystine uptake in system Xc^-^. It mainly includes erastin and its derivatives (such as imidazole ketone erastin, perazine erastin), sulfasalazine, sorafenib, etc. 2) *FIN associated with GPX4.* These inducers can be further subdivided into three kinds: a) directly blocking GPX4 enzyme activity, including RSL3, ML162, ML210, DPI7, etc.; b) depleting GPX4 protein, binding squalene synthase (SQS) and depleting antioxidant CoQ10, such as FIN56; c) directly oxidizing Fe^2+^ and indirect inactivating GPX4, such as FINO2 (90, 98).

According to reports, there are mainly 14 compounds (such as erastin, RSL3, talaroconvolutin-A, etc.) that can be used in CRC treatment (**Table 1**). RSL3 is a classical inhibitor by directly inhibiting the catalytic activity of GPX4. It utilizes the electron-philic chloroacetamide fraction to covalently bind to the selenocysteine residue of GPX4, which promotes the accumulation of intracellular ROS and lipid peroxidation, thereby triggering ferroptosis (99). Cetuximab inhibits the Nrf2/HO-1 axis by activating p38 MAPK and enhances RSL3-induced ferroptosis (111). Erastin was the first FIN to be discovered. By binding with SLC7A11, erastin can inhibit its activity and affect cystine transport, thereby reducing GSH synthesis, which results in the failure of removing lipid peroxides in time, then causing cell membrane damage and triggering ferroptosis (112). Talaroconvolutin-A (TalaA) is a novel ferroptosis inducer, which is more effective than erastin according to report. TalaA can promote ferroptosis through not only promoting ROS production but also down-regulating SLC7A11 and up-regulating arachidonate lipoxygenase 3 (ALOXE3) (103). Lysionotin (Lys) is a flavonoid compound that promotes the accumulation of ROS in CRC cells and increases the degradation rate of Nrf2 protein, resulting in ferroptosis (113). Petunidin 3-O-[rhamnopyranosyl-(trans-p-coumaroyl)]-5-O-(β-D-glucopyranoside) (Pt3R5G) further inhibits the proliferation of human colonic adenocarcinoma cells (RKO) primarily by down-regulating SLC7A11 to inhibit ferroptosis (114). Auriculasin promotes CRC apoptosis, ferroptosis, and oxidative apoptosis by inducing ROS production, thereby inhibiting cell infiltration (114). Double-targeted PI3K and HDAC inhibitor BEBT-908 developed by Fan et al. can induce cancer cell ferroptosis and effectively inhibit tumor cell growth by up-regulating MHC class I molecule of tumor cell and activating endogenous IFN- γ signal through STAT1 signaling pathway (115). Tagitinin C induces ferroptosis through endoplasmic reticulum stress-mediated activation of the PERK-Nrf2-HO-1 signaling pathway (110).

**Table 1:** Ferroptosis inducers for CRC treatment.

Drug/compound	Mechanisms	References
RSL3	Inhibited GPX4	(99)
Resibufogenin	Inhibited GPX4	(100)
Erastin	Inhibited SLC7A11	(101)
Benzopyran derivative 2-imino-6-methoxy-2H-chromene-3-carbothioamide (IMCA)	Down-regulated SLC7A11	(101)
Paclitaxel	Down-regulated SLC7A1 and up-regulated p53	(102)
Oxaliplatin	Inhibited NRF2	(95)
Talaroconvolutin A	Promoted ROS production, down-regulated SLC7A11, up-regulated ALOXE3	(103)
β-elemene	Combined with cetuximab to promoted ROS production, up-regulated HO-1 and transferrin, down-regulated of GPX4, SLC7A11 in KRAS mutant CRC cells	(104)
Vitamin C	Promoted ROS production and combined with cetuximab for anti-EGFR therapies in CRC	(105)
Bromelain	Promoted ROS production by up-regulating ACSL4 in KRAS-mutant CRC cells	(106)
Sulfasalazine	Enhanced sensitivity of cisplatin to CRC by inhibiting system Xc^-^	(107)
Sorafenib	Induced lipid peroxidation and adjusted iron metabolism	(108)
Artemisinin and its derivatives (Artesunate, dihydroartemisinin)	Adjusted iron metabolism by targeting ferritinophagy	(109)
Tagitinin C	Up-regulated HO-1 and promoted lipid peroxidation	(110)

Since the concept of ferroptosis was proposed in 2012, the initial design ideas of some drugs like those listed in **Table 1** were not based on the mechanism of ferroptosis. For example, oxaliplatin exerted anti-tumor effect mainly by inhibiting DNA replication and transcription. It produced biologically active hydrated derivatives in body fluids that form intra- and inter-strand crosslinks with DNA (116). However, its ferroptosis-inducing effect has been found in recent studies. At present, there is no clear evidence that oxaliplatin has a more obvious effect on inhibiting DNA synthesis or inducing ferroptosis. However, according to the existing research results, ferroptosis is an effective adjuvant treatment method at least, which can enhance the overall treatment effect (117).

In addition to the drugs mentioned above, some other proteins and enzymes have extensive effects, including miR-19a, OTUD1, miR-15a-3p, HSPA5 and N-acetyltransferase 10 (NAT10), etc., which can also be involved in the regulation of iron death and thus affect the occurrence and development of colorectal cancer. For example, oncogenic miR-19a negatively regulates ferroptosis inducer iron responsive element binding protein 2 (IREB2), inhibiting the growth of CRC cells and reducing the risk of ferroptosis (118). OTUD1 is a deubiquitinating enzyme of IREB2 that stabilizes IREB2-mediated iron transport, leading to increased ROS production and ferroptosis (119). Overexpression of miR-15a-3p can inhibit GPX4, resulting in increased levels of ROS, intracellular Fe^2+^ and malondialdehyde (120). HSPA5 protects cells against ferroptosis, which promotes the occurrence and development of CRC by maintaining the stability of GPX4 and inhibiting ferroptosis (121). N-acetyltransferase 10 (NAT10) can affect the mRNA stability and expression of ferroptosis suppressor protein 1 (FSP1). The overexpressed of NAT10 can enhance the cells proliferation, migration, invasion, tumorigenesis and metastasis, and reduce the patient survival time (122).

#### 4.2.2 Nanomedicine based on ferroptosis

Nanotechnology has been extensively studied to develop advanced nanoparticle drug delivery systems for anticancer drug delivery, which has significant advantages in improving drug availability and targeted delivery properties. The disadvantages of low solubility and poor membrane permeability can be well overcome by preparing anticancer drugs into nanoparticles (123). More importantly, nanomedicine has unique advantages in cancer treatment due to the enhanced permeability and retention (EPR) effect and easy surface modification of nanoparticles (124). Recently, there has been increasing interest in ferroptosis driven nanomedicine due to the potent antitumor activity offered by the combination of ferroptosis and nanotechnology (125). Pan et al. reported that zinc oxide nanoparticles could inhibit GSH synthesis by scavenging H_2_S from CRC to induce ferroptosis (126). Li et al. designed a nanoplatform (GCMNPs) based on glycyrrhetinic acid (GA)/poly (lactic-co-glycolic acid), which could induce ferroptosis by increasing intracellular H_2_O_2_, Fe^2+^ and lipid peroxidation levels and suppressing GPX4 expression. Moreover, they confirmed that combination of GCMNPs and ferrotherapy could enhanced Fenton reaction and immune response in CRC mouse models (127). Chen et al. constructed nanoelicitors, which included two immune-elicitable polyphenols (Chlorogenic acid and Mitoxantrone) and Fe^3+^ ions. The results showed that nanoelicitors could activate tumoricidal immunityto enhance ferroptosis in CRC treatment (128). Han et al. reported core-shell structure nanoparticles of ZnP@DHA/Pyro-Fe, which was used to co-deliver dihydroartemisinin (DHA) and pyropheophorbide-iron (Pyro-Fe). This strategy could remarkably enhance ferroptosis and therapeutic effect of DHA in CRC mouse models (128). Carbon dots (CDs) are zero-dimensional nanomaterials. Tian et al. prepared pentacyclic triterpenes (PTs), a natural anticancer product, into PTs-CDs, which can induce and selectively kill cancer cells by targeting apoptosis, autophagy and ferroptosis of tumor mitochondria (129).

As mentioned above (**Table 2**), there are few reports on the nanoparticles by promoting ferroptosis for CRC treatment. Moreover, due to poor effect of monotherapy in tumor treatment, the combination of ferroptosis with tumor targeted imaging, phototherapy, chemotherapy, autophagy or immune regulation is going to be a new trend. Therefore, the nanomedicine based on ferroptosis for CRC treatment has great space for development.

**Table 2:** Nanoparticles based on ferroptosis for CRC treatment.

Nanoparticles	Components	Mechanisms	References
Zinc oxide nanospheres	ZnO; Virus-like mesoporous silica nanoparticles;	Inhibited GSH synthesis by scavenging H_2_S	(126)
Glycyrrhetinic acid nanoparticles	Glycyrrhetinic acid; Poly (lactic-co-glycolic acid); Leukocyte membrane; Ferumoxytol	Inhibited GPX4 Increasd intracellular H_2_O_2,_ Fe^2+^ and lipid peroxidation levels Enhanced Fenton reaction by introducing exogenous Fe iron	(127)
Self-assembly nanoelicitors	Chlorogenic acid (CA); Mitoxantrone (MIT); Fe^3+^ ions; PEG-AZB-O; HSPC; Cholesterol	CA and MIT induced tumoricidal immunity and promoted the cytotoxic T lymphocyte to release IFN-γ, which could inhibit system Xc^-^ to GPX4 pathway. Enhanced Fenton reaction by introducing exogenous Fe iron.	(128)
ZnP@DHA/Pyro-Fe nanoparticles	Zin phosphates; DOPA; DOPC; DSPE-PEG2k; Cholesterol; Cholesterol derivative of dihydroartemisinin (Chol-DHA); pyropheophorbide-iron (Pyro-Fe);	Enhanced Fenton reaction by introducing exogenous Fe iron	(128)

## 5 Summary and prospect

This mini review summarizes the epidemiology, symptoms, etiology and pathogenesis of CRC, focusing on three molecular mechanisms (CIN, MSI and CIMP) of CRC. The basic characteristics, mechanisms of ferroptosis and its potential association with CRC are also summarized. The mechanisms including lipid metabolism, amino acid metabolism, iron metabolism and other metabolic methods that affect the occurrence and execution of ferroptosis are highlighted. In addition, the research status of FIN and nanomedicine in CRC treatment are emphatically introduced.

In recent years, although significant progress has been made in the research of ferroptosis in cancer treatment, it is still in the initial stage, mainly focusing on basic research. However, the mechanism between ferroptosis and CRC remains unclear. Whether there are specific regulatory factors or signaling pathways. Ferroptosis and other forms of programmed cell death, which have a greater impact on CRC, require further research and confirmation.

The ultimate goal of research is to achieve clinical transformation and benefit patients. But there is still a long way to go before clinical application, and there are still many problems to be solved. Which patients are more likely to benefit from ferroptosis-related treatment. Which gene or protein can be used as biomarkers for patients’ response to ferroptosis treatment. What are the adverse effects of ferroptosis treatment and how to control adverse reactions. There is still no clear answer to the above question, which needs further exploration.

Although ferroptosis faces many difficulties and challenges on the road to clinical application, as an emerging cell death type, ferroptosis-based therapeutic strategies still have great potential. Although there are few studies on nanoparticles involving ferroptosis for CRC treatment, the combination of ferroptosis with nanotechnology and various theranostic modalities is inevitable development trend. Overall, ferroptosis promises to open a new door for cancer treatment including CRC.

## Author contributions

HL, XH and YT wrote the manuscript. NB, YP and KH prepared tables and figures. YW designed and reviewed the manuscript. All authors contributed to the article and approved the submitted version.
